# Supplementary figures and images for: Widespread dynamic and pleiotropic expression of the melanocortin‐1‐receptor (MC1R) system is conserved across chick, mouse and human embryonic development
Source: Birth Defects Res. 2018 Jan 8;110(5):443–55. doi: 10.1002/bdr2.1183 (PMC6446732; doi:10.1002/bdr2.1183)

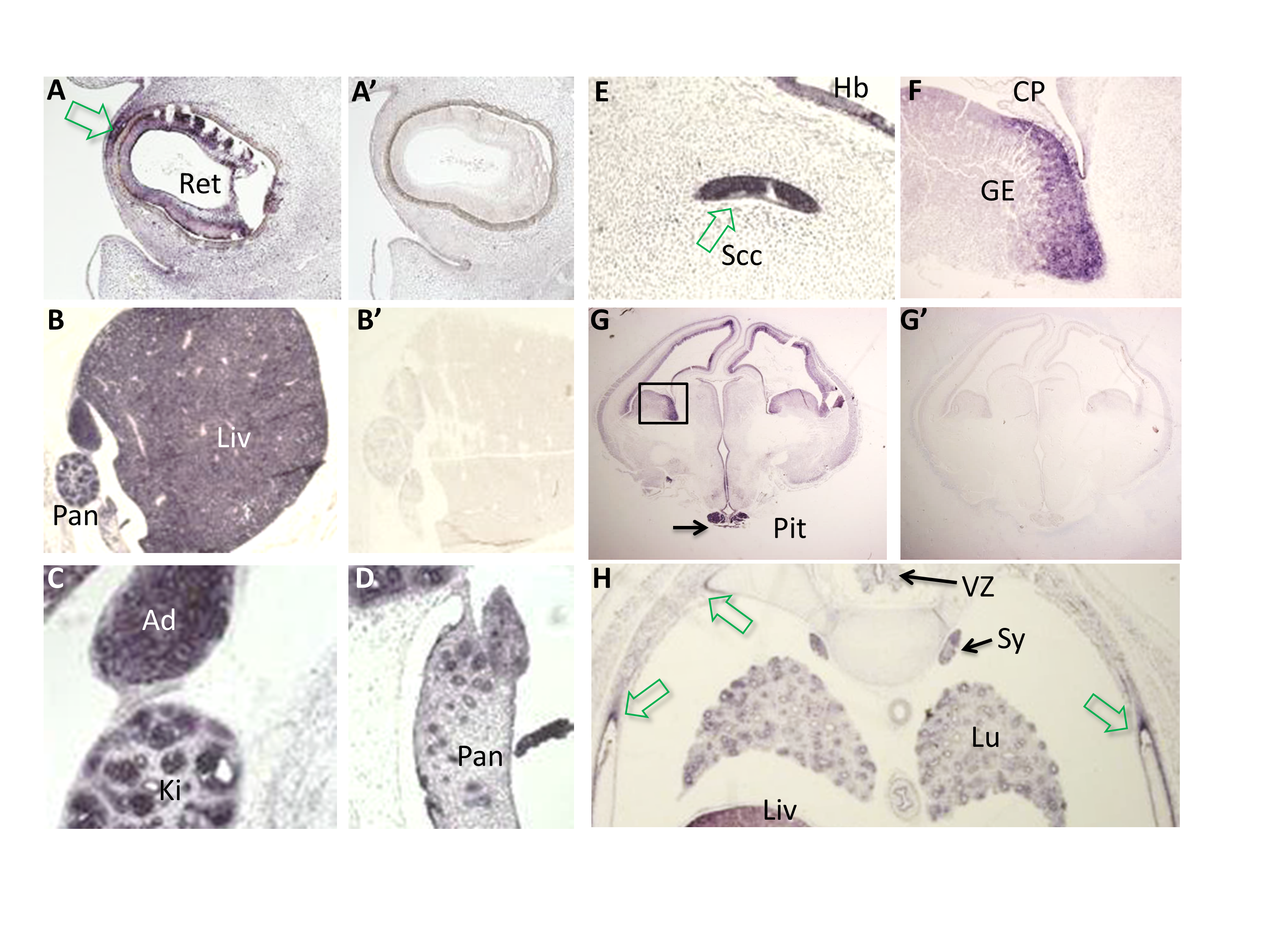

Supplement: Supplementary file 1 — Supporting Information [file BDR2-110-443-s001.tif]
